# Biological function of a polysaccharide degrading enzyme in the periplasm

**DOI:** 10.1038/srep31249

**Published:** 2016-11-08

**Authors:** Yajie Wang, M. Fata Moradali, Ali Goudarztalejerdi, Ian M. Sims, Bernd H. A. Rehm

**Affiliations:** 1 Institute of Fundamental Sciences, Massey University, Palmerston North, New Zealand; 2 Department of Pathobiology, School of Paraveterinary Science, Bu-Ali Sina University, Hamadan, Iran; 3 The Ferrier Research Institute, Victoria University of Wellington, Lower Hutt, Wellington, New Zealand; 4 MacDiarmid Institute for Advanced Materials and Nanotechnology, Massey University, Palmerston North, New Zealand

## Abstract

Carbohydrate polymers are industrially and medically important. For instance, a polysaccharide, alginate (from seaweed), is widely used in food, textile and pharmaceutical industries. Certain bacteria also produce alginate through membrane spanning multi-protein complexes. Using *Pseudomonas aeruginosa* as a model organism, we investigated the biological function of an alginate degrading enzyme, AlgL, in alginate production and biofilm formation. We showed that AlgL negatively impacts alginate production through its enzymatic activity. We also demonstrated that deletion of AlgL does not interfere with polymer length control, epimerization degree or stability of the biosynthesis complex, arguing that AlgL is a free periplasmic protein dispensable for alginate production. This was further supported by our protein-stability and interaction experiments. Interestingly, over-production of AlgL interfered with polymer length control, suggesting that AlgL could be loosely associated with the biosynthesis complex. In addition, chromosomal expression of *algL* enhanced alginate O-acetylation; both attachment and dispersal stages of the bacterial biofilm lifecycle were sensitive to the level of O-acetylation. Since this modification also protects the pathogen against host defences and enhances other virulence factors, chromosomal expression of *algL* could be important for the pathogenicity of this organism. Overall, this work improves our understanding of bacterial alginate production and provides new knowledge for alginate production and disease control.

Carbohydrate polymers are produced by organisms within all domains of life, serving roles in structure, energy storage, and signalling. Structurally diverse and composed of a plethora of monomeric building blocks, many are of ecological, industrial and medical significance^1^,^2^,^3^. For instance, a polysaccharide, alginate, harvested from seaweed, is widely used in food, textile and pharmaceutical industries owing to its viscosifying and gelling properties^4^,^5^. However, due to seasonal variations in molecular weight and composition, obtaining alginate of defined properties from seaweed for high-value medical-applications is often costly and difficult. Alginate is also produced by two bacterial genera, soil dwelling *Azotobacter* and ubiquitous *Pseudomonas*. Previous research have shown that, unlike seaweed, bacteria such as *Azotobacter vinelandii* and *Pseudomonas aeruginosa* produce highly homogenous alginate^4^. The former species has been trialled for manufacturing alginates of defined properties^6^,^7^ while the latter species over-produces alginate during cystic fibrosis (CF) lung infections, allowing it to evade host immunity and antibiotics^5^,^8^. Therefore, better understanding of bacterial alginate biosynthesis mechanisms may allow production of tailor-made alginates for high-value medical applications and help combat bacterial infections.

Alginate is composed of *β*-_D_-mannuronic acid (M) and its C5 epimer, *α*-_L_-guluronic acid (G), linked by *β*-1,4 glycosidic bonds. In bacteria, it is synthesized by a synthase dependent mechanism involving an envelope spanning multi-protein complex, which is encoded within the alginate biosynthesis gene cluster (*algD*, *8*, *44*, *K*, *E*, *G*, *X*, *L*, *I*, *J*, *F*, *A*). The alginate precursor, GDP-mannuronic acid, is synthesized from the central metabolite fructose-6-phosphate in four-steps by AlgA, AlgC and AlgD^9^. This precursor is polymerized by the alginate polymerase Alg8, forming poly-mannuronate (poly-M) which is transported across the inner membrane (IM)^10^,^11^,^12^. This requires binding of bis-(3′-5′)-cyclic dimeric guanosine monophosphate (c-di-GMP), a bacterial secondary messenger, to the PilZ domain of the alginate co-polymerase Alg44^13^,^14^,^15^. After crossing the IM, poly-M is transported through the periplasm, the space between the inner and outer membranes (OM), by a membrane spanning multi-protein machine (Alg44, X, G, K)^14^,^16^,^17^ to the OM protein, AlgE, for secretion^18^,^19^,^20^. In the periplasm, poly-M is modified by O-acetylation (AlgI, J, F and X)^21^,^22^,^23^ and epimerization (AlgG)^24^,^25^,^26^. These modifications add acetyl groups to the C-2 and/or C-3 positions of M residues^21^,^22^ and convert M residues into G residues^24^,^26^, respectively. Both modifications affect polymer composition and its barrier properties against therapeutic intervention and host immunity^14^,^27^,^28^,^29^.

Intriguingly, an alginate degrading enzyme, AlgL, is also encoded within the alginate biosynthesis gene cluster. Although several efforts have been made to investigate the role of AlgL in alginate production, it has been difficult to draw conclusions due to inconsistent findings. For instance, deletion of *algL* either had no effect^30^, significantly reduced^31^,^32^ or completely abolished alginate production^33^,^34^,^35^,^36^ in different species (strains) of alginate producing bacteria. Furthermore, there is only limited information on the effect of AlgL on alginate polymer length^32^,^33^,^37^ and degree of epimerization^33^, and so far nothing is known about the effect of AlgL on alginate o-acetylation. Furthermore, although Moradali *et al*.^14^ showed that the molecular mass of alginate was reduced by epimerization, while it was increased by acetylation, the role of AlgL and its lyase activity in controlling polymer length, composition and alginate yield is still unknown.

Even though the exact biological function of AlgL remains undetermined, two preliminary models for its role in alginate biosynthesis have been proposed^33^,^36^. These models suggest that AlgL undertakes a maintenance role in the periplasm by degrading alginate that has been misguided due to inefficient translocation/secretion, deletion of a subunit or destabilization of the complex. However, they disagree on whether AlgL is part of the biosynthesis apparatus. For instance, while Bakkevig *et al*.^33^ suggested that AlgL only served a maintenance role (thus is likely to be a free periplasmic protein), Jain and Ohman^36^ postulated that it also facilitated translocation and secretion of the polymer, therefore forming a structural component of the multi-protein complex. However, evidence for protein-protein interactions between AlgL and other components of the complex was still lacking. To test the validity of these models, we performed protein stability and interaction experiments to determine whether or not AlgL was part of the biosynthesis complex.

*P. aeruginosa* and its over-production of alginate are used as a model system for understanding biofilms which are a major challenge in medical and industrial settings^38^,^39^. Arguably two of the most important stages of biofilm lifecycle are attachment and dispersal. During attachment, cells adhere to a surface through appendages and surface-associated polysaccharides, allowing cells to initiate biofilm formation, while during the dispersal stage free-living cells are released from mature biofilms by matrix-degrading enzymes, allowing them to colonize new surfaces^40^. In spite of alginate being a major component of *P. aeruginosa* biofilms, its role in attachment remains controversial. For example, some support a function for alginate in attachment^41^ while others reject it^42^,^43^. Similarly, the effect of alginate degrading enzymes on dispersal remains poorly understood. Although some researchers proposed that alginate lyase activity (provided as an extracellular enzyme) enhanced cell release^43^, others asserted that dispersal was not dependent on alginate lyase activity^44^. However, the effect of endogenously produced alginate lyase on biofilm lifestyle remains poorly characterized. Therefore, a better understanding of biological function of alginate and AlgL in biofilm attachment and dispersal would help inform future strategies utilizing alginate degrading enzymes for combating *P. aeruginosa* infections.

In this study, we used *P. aeruginosa* as a model organism to investigate the biological function of an alginate degrading enzyme, AlgL, in modulating alginate yield, molecular weight and composition. Through protein-stability and interaction experiments, we also examined whether AlgL was a free periplasmic protein or a subunit of the alginate biosynthesis apparatus. Thirdly, we assessed the effect of deleting *algL* on biofilm attachment and dispersal through microtiter plate assays. Overall, the knowledge generated in this study is of importance for development of approaches for homogenous alginate production and disease treatment.

## Results

### AlgL negatively impacts alginate yield

**Figure 1:**
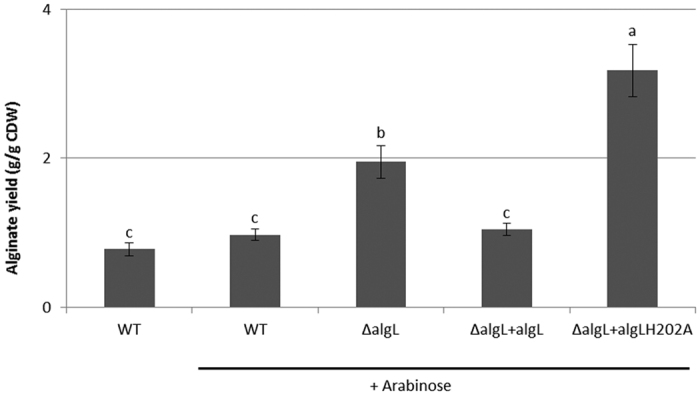
Alginate yield. Mean alginate yields ± standard error for strains grown on PIA medium (containing 300 μg/ml of Carbenicillin) in presence (+) of arabinose inducer (0.5% w/v). Mean alginate yields are presented as grams of alginate produced per gram of cellular dry weight (g/g CDW). Different letters displayed above columns indicate statistically significant difference (p < 0.05) in alginate yield across treatments as determined by ANOVA followed by an LSD multiple comparison analysis (n = 3). Strains are identified as follows: WT = PDO300(pHERD20T); ΔalgL = PDO300Δ*algL*(pHERD20T); ΔalgL + algL = PDO300Δ*algL*(pHERD20T:*algL*), and ΔalgL + algLH202A = PDO300Δ*algL*(pHERD20T:*algL*_*H202A*_).

To elucidate the role of AlgL in alginate production, the *algL* gene was disrupted in an alginate over-producing strain, *P. aeruginosa* PDO300. While the wild type (WT) strain harboring an empty vector, PDO300(pHERD20T), produced 0.78 ± 0.09 grams of alginate per gram of cellular dry weight (g/g CDW ± standard error), its *algL* mutant, PDO300Δ*algL*(pHERD20T), produced significantly more alginate (1.95 ± 0.22 g/g CDW) (Fig. 1). Over-expressing the *algL* gene from an arabinose-inducible promoter on the plasmid pHERD20T:*algL* in PDO300Δ*algL*(pHERD20T:*algL*) restored alginate yield to WT levels (1.04 ± 0.08 g/g CDW) (Fig. 1). In contrast, when a catalytically inactive variant of *algL* was overexpressed in the *algL* mutant, PDO300Δ*algL*(pHERD20T:*algL*_*H202A*_), alginate yield was increased by three-fold (to 3.18 ± 0.35 g/g CDW) compared to WT strain harboring an empty vector, PDO300(pHERD20T). Taken together, our findings suggest that AlgL negatively impacts alginate production in a manner dependent on its enzymatic activity.

As expected, the AlgL protein was absent in the cell lysates of the *algL* mutant and present in lysates of PDO300Δ*algL*(pHERD20T:*algL*) (Fig. S1).

### Over-production of AlgL interferes with polymer length control

**Figure 2:**
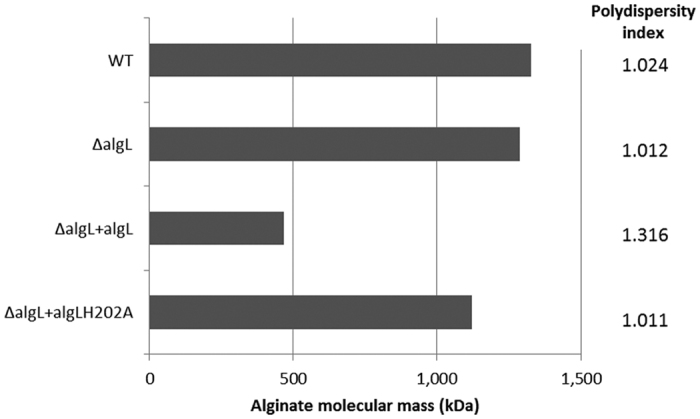
Alginate molecular weight (MW) averages and polydispersity indices as determined by SEC-MALLS. Alginate samples from strains grown on PIA medium containing 300 μg/ml of Carbenicillin and 0.5% (w/v) arabinose inducer were analysed by SEC-MALLS to determine their average molecular mass and polymer length distribution (polydispersity index = PI). PI values closer to 1.0 represent a narrow M_r_ distribution (i.e. homogenous M_r_). Strains are identified as follows: WT = PDO300(pHERD20T); ΔalgL = PDO300Δ*algL*(pHERD20T); ΔalgL + algL = PDO300Δ*algL*(pHERD20T:*algL*), and ΔalgL + algLH202A = PDO300Δ*algL*(pHERD20T:*algL*_*H202A*_).

To examine whether AlgL modulated alginate polymer length, samples were analyzed by size exclusion chromatography-multi-angle laser light scattering (SEC-MALLS) and their average molecular mass (M_r_) and polydispersity index (PI) were determined. PI values of 1.0 indicated a narrow M_r_ range (i.e. homogenous polymer length distribution) while PI values greater than 1.0 implied a heterogeneous distribution. Here, we demonstrated that both the WT strain and its *algL* mutant, PDO300(pHERD20T) and PDO300Δ*algL*(pHERD20T), produced alginates with similar molecular mass averages (around 1,300 kDa) and PIs (about 1.020) (Fig. 2), indicating that deletion of *algL* did not impact on alginate molecular weight. However, plasmid-borne expression of active AlgL in the *algL* mutant, PDO300Δ*algL*(pHERD20T:*algL*), led to a lower molecular mass (M_r_: 466 kDa) and a substantially wider MW distribution (PI value of 1.3) (Fig. 2), indicating that when AlgL was over-produced, it interfered with polymer length control. This effect appeared to be dependent on the catalytic activity of AlgL, since over-production of catalytically inactive AlgL_H202A_ in the *algL* mutant, PDO300Δ*algL*(pHERD20T:*algL*_H202A_), yielded alginates with average molecular masses (1,122 kDa) and PI values (1.011) closer to that of WT and *algL* mutant (Fig. 2).

In principle, excess AlgL could have affected polymer length through periplasmic and/or extracellular degradation of the polymer. However, strains over-producing AlgL did not possess any extracellular lyase activity as demonstrated by an alginate degradation plate assay (Fig. S2). Thus, given the periplasmic localization of AlgL^33^,^36^, we propose that when active AlgL is over-produced it influences alginate molecular mass from within the periplasm through its enzymatic activity.

### Chromosomal expression of *algL* is critical for efficient O-acetylation

The *algL* gene is immediately flanked by genes (*…G*, *X*, ***L***, *I*, *J*, *F…*) involved in alginate epimerization (*algG*) and O-acetylation (*algX*, *I*, *J*, *F*). The position of the *algL* gene in this operon led us to hypothesis that AlgL could affect alginate composition. To this end, we analyzed the composition of alginate samples produced by the WT strain [PDO300(pHERD20T)], *algL* mutant [PDO300Δ*algL*(pHERD20T)], and its complemented strains expressing active [PDO300Δ*algL*(pHERD20T:*algL*)] and inactive [PDO300Δ*algL*(pHERD20T:*algL*_*H202A*_)] variants of AlgL, using ^1^H-nuclear magnetic resonance (^1^H-NMR) spectroscopy. Our results demonstrate that all four strains produced alginates with similar levels of epimerization (*F*_*G*_: 0.32 ± 0.04, Table 1), indicating that epimerization degree is not substantially affected by AlgL copy number or its catalytic activity. As expected, none of these strains produced alginates with GG blocks (Table 1), confirming previous reports regarding the lack of GG blocks in alginates produced by members of the *Pseudomonas* genera^45^.

However, when comparing alginate O-acetylation levels, the WT strain produced alginates with significantly higher levels of O-acetylation (Ac: 70%) than that of the *algL* mutant and its complemented strains (Ac: 17–20%, Table 1), strongly suggesting that chromosomal expression of AlgL was necessary for efficient O-acetylation.

### AlgL is a free periplasmic protein dispensable for alginate production

Two models had been proposed for the role of AlgL. Although both models assigned a maintenance role to AlgL in degrading misguided alginate in the periplasm, they disagreed if AlgL was a free periplasmic protein or a subunit of the multi-protein complex. To clarify this, we examined the effect of deleting *algL* on the stability of various components of the multi-protein apparatus, and also explored whether deleting various components of the complex also affected AlgL stability.

We reasoned that if AlgL was a free-periplasmic protein dispensable for alginate production, then (i) its absence (by deletion of the *algL* gene) would not compromise alginate production or (ii) the stability of the biosynthesis machinery. Furthermore, (iii) removal of structural components of the multi-protein biosynthesis complex such as Alg44, K, X and E (by knocking out their respective genes) would also not affect AlgL stability.

**Figure 3:**
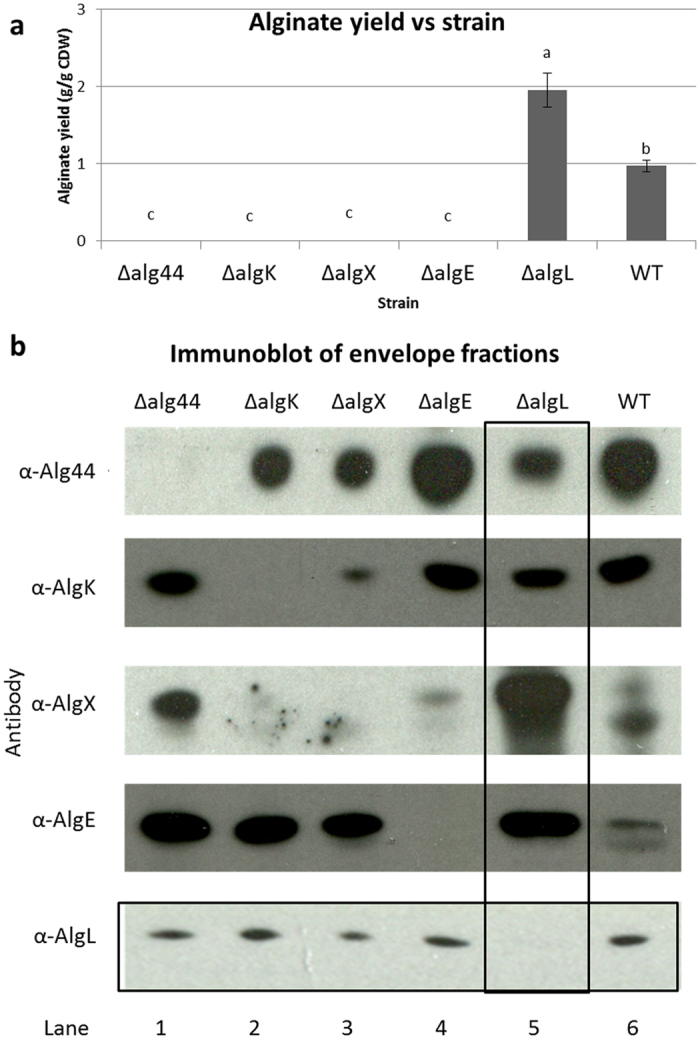
Alginate yield and alginate biosynthesis machinery stability in various mutants during growth on solid medium. (**a**) Mean alginate yield presented as grams of alginate per gram of cellular dry weight (g/g CDW) ± SE. Data in panel (**a**) were not normally distributed (Shapiro-Wilk test: P < 0.05), and thus analysed using non-parametrical ANOVA followed by Bonferroni (Dunn) *t* tests for multiple comparison between treatments (n = 3). Different letters indicate statistically significant differences (p < 0.05) in alginate yields across treatments. (**b**) Biosynthesis apparatus stability. Shown are immunoblots of envelope fractions of various strains using anti-Alg antibodies (left panel: α-Alg44, α-AlgK, α-AlgX, α-AlgE and α-AlgL) to detect specific components of biosynthesis complex. Vertical box highlights impact of deleting AlgL on apparatus stability while horizontal box reveals effect of deleting various apparatus subunits on AlgL stability. In panels (**a** and **b**), strains are identified as follows: Δalg44 = PDO300Δ*alg44*, ΔalgK = PDO300Δ*algK*, ΔalgX = PDO300Δ*algX*, ΔalgE = PDO300Δ*algE*, ΔalgL = PDO300Δ*algL*, and WT = *P. aeruginosa* PDO300. Full-length immunoblots are presented in Supplementary Fig. S3.

In agreement with this scenario, we revealed that during growth on solid medium, (i) deletion of *algL* in PDO300 did not impair alginate production (Fig. 3a) or (ii) the stability of the complex (Fig. 3b). In particular, deletion of *algL* did not affect localization of Alg44, K, X or E to the envelope fraction (vertical box, Fig. 3b). Furthermore, (iii) removal of other subunits (Alg44, K, X or E) did not affect AlgL localization to the membrane fraction (horizontal box, Fig. 3b). These results indicated that AlgL stability was independent of the stability of the complex, implying that AlgL could be a free periplasmic protein dispensable for alginate production.

**Figure 4:**
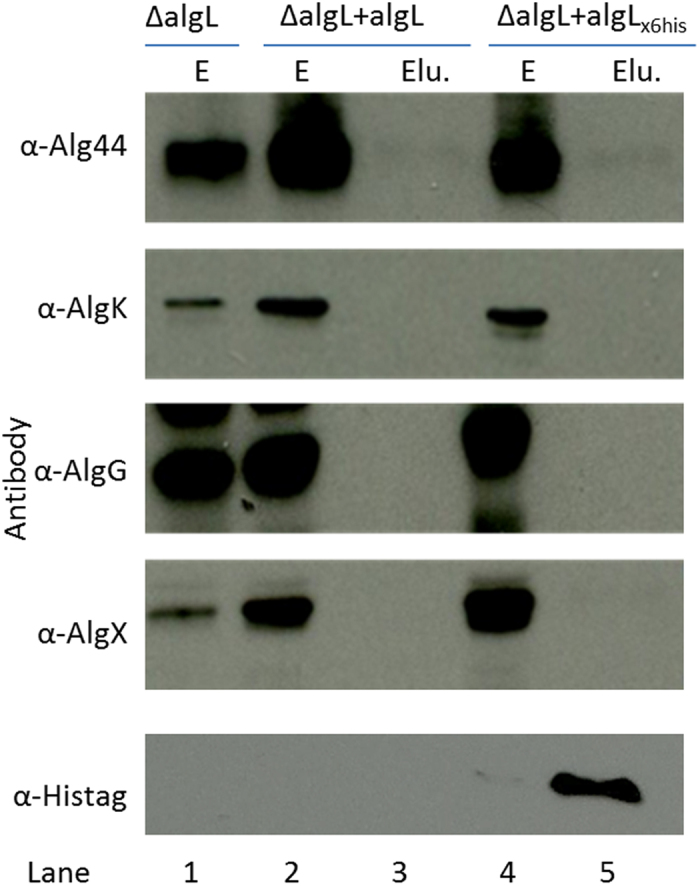
Pull down and immunoblot experiment using hexahistidine-tagged AlgL. Strains were grown on PIA medium supplemented with 300 μg/ml of Carbenicillin (except for PDO300Δ*algL*, lane 1) and 0.05% (w/v) arabinose for 72 h at 37 °C. Envelope (E) fractions were prepared and histagged-AlgL and its potential co-interacting proteins were purified with cOmplete His-Tag Purification Resin (Roche). Envelope (E) and eluted (Elu.) fractions were run on SDS-PAGE, transferred to nitrocellulose membrane and subject to immunoblot with various antibodies (left panel: α-Alg44, α-AlgK, α-AlgG, α-AlgX and α-Histag), as described in methods section. Lane 1 = Envelope fraction of PDO300Δ*algL*. Lanes 2 and 3: Envelope (E) and eluted (Elu.) fractions of PDO300Δ*algL*(pHERD20T:*algL*), respectively. Lanes 4 and 5: Envelope (E) and eluted (Elu.) fractions of PDO300Δ*algL*(pHERD20T *algL*_*x6his*_), respectively. Full-length immunoblots are presented in Supplementary Fig. S4.

To further confirm this finding, we performed pulling down experiments and immunoblotting using hexahistidine-tagged AlgL produced in PDO300Δ*algL*(pHERD20T:*algL*_*6xhis*_). Strains were grown on solid medium and his-tagged-AlgL was purified from solubilized envelope fractions by his-affinity chromatography under native conditions. Eluted fractions were probed with various antibodies (anti-Alg44, -AlgK, -AlgX and -AlgG) to detect potential interacting partners. While his-tagged AlgL was detected in the eluted fraction with anti-his antibodies, none of the following subunits of the biosynthesis complex (Alg44, AlgK, AlgG or AlgX) were detectable in the eluted fractions (Fig. 4), providing additional evidence that AlgL is likely to be a free periplasmic protein.

### Effect of AlgL on biofilm lifestyle

**Figure 5:**
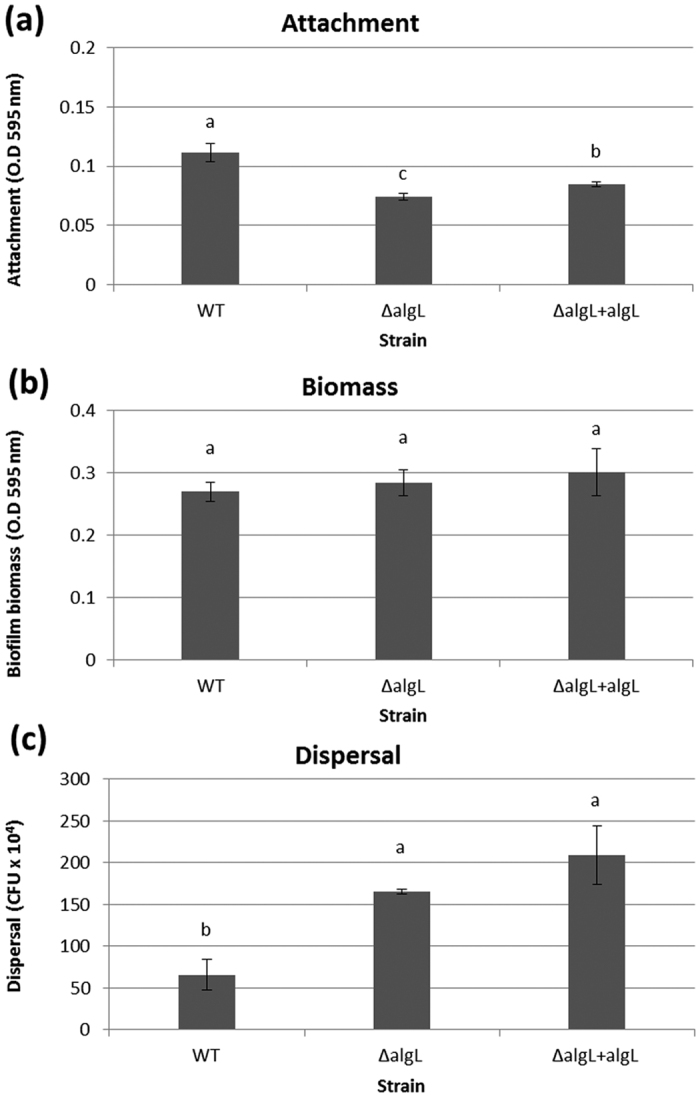
Effect of AlgL on cell attachment, biofilm biomass and dispersal. Attachment efficiencies, biofilm biomasses and dispersal efficiencies were analyzed in a microtiter plate assay. (**a**) Mean attachment efficiencies ± SE (n = 8) at 2 h, as determined by crystal violet staining. Attachment efficiencies are expressed as absorbances at 595 nm. (**b**) Mean biomass of biofilms ± SE (n = 4) at 72 h, as determined by crystal violet staining. (**c**) Mean dispersal efficiencies ± SE (n = 4) at 72 h expressed as colony forming units (CFU × 10^4^). In panels (**a**–**c**) different letters displayed above columns indicate statistically significant differences (p < 0.05) in attachment efficiency, biofilm biomass and dispersal efficiency, respectively. Attachment efficiencies (**a**) were not normally distributed (Shapiro-Wilk test: P < 0.05) and thus were analyzed using non-parametrical ANOVA followed by Bonferroni (Dunn) *t* tests for multiple comparison between treatments. Biofilm biomasses (**b**) and dispersal efficiencies (**c**) were normally distributed (Shapiro-Wilk test: P > 0.05), and thus analyzed using ANOVA with multiple LSD test for multiple comparison between treatments. In panels (**a**–**c**), strains are identified as follows: WT = PDO300(pHERD20T), ΔalgL = PDO300Δ*algL*(pHERD20T), and ΔalgL + algL = PDO300Δ*algL*(pHERD20T:*algL*).

To examine whether AlgL has a more general function in the biofilm lifestyle of *P. aeruginosa*, we performed cell surface attachment and dispersal assays in 96 well microtiter plates. Our results showed that the mean attachment efficiencies (±standard error) after 2 h of biofilm establishment for WT [PDO300(pHERD20T)], *algL* mutant [PDO300Δ*algL*(pHERD20T)] and complemented strain [[PDO300Δ*algL*(pHERD20T:*algL*)] were 0.111 ± 0.008, 0.074 ± 0.003 and 0.085 ± 0.002, respectively (Fig. 5a). Subsequent statistical analysis indicated that the WT strain exhibited significantly greater attachment efficiency than the *algL* mutant and its complemented strain (Fig. 5a). Interestingly, greater attachment efficiency of the WT strain (Fig. 5a) was linked to higher O-acetylation levels (Ac: of 70% for WT strain vs Ac: of 17–20% for latter strains), suggesting that O-acetylation influences cell attachment. However, no link between attachment efficiency and alginate yield or polymer length was observed.

At later stages of the biofilm lifecycle (by 72 h), all three strains reached similar biomasses (Fig. 5b), indicating that AlgL, alginate yield, molecular mass and composition did not impact on the biomass reached by biofilms after three days of growth. Subsequent dispersal assays performed at this later time revealed that the cell dispersal of the WT strain was less efficient than that of the other two strains (Fig. 5c). Since the above results (Fig. 5) suggested an effect of O-acetylation degree on attachment and dispersal, we carried out more experiments to assess if O-acetylation directly influenced these phenotypes. To this end, we performed similar attachment and dispersal assays in microtiter plates using two previously described strains, PDO300Δ*algX*(pBBR1MCS-5:*algX*) and PDO300Δ*algX*(pBBR1MCS-5:*algXS269A*), which produced O-acetylated (Ac: 10%) and non-o-acetylated (Ac: 0%) forms of alginate^14^, respectively. We used these strains for better control because both strains produced alginates of comparable yield, mass, length and epimerization degree. Furthermore, the former strain produced a catalytically active terminal acetyl-transferase responsible for O-acetylation (AlgX) while the latter only expressed catalytically inactive AlgXS269A. Our results demonstrate that abolishing O-acetylation significantly impaired attachment and dispersal without affecting biofilm biomass (Fig. S5). Based on these results, we suggest that both attachment and dispersal stages of the *P. aeruginosa* biofilm lifecycle are sensitive to alginate O-acetylation levels.

## Discussion

Using *P. aeruginosa* as a model organism, we examined the biological function of a polysaccharide degrading enzyme, AlgL, in modulating alginate production, molecular mass and composition. Previously, several groups investigated the role of AlgL in alginate biosynthesis by generating *algL* mutants in various alginate producing bacteria. However, inconsistencies made it difficult to draw conclusions. In contrast to earlier studies, the *algL* mutant generated in this report produced twice as much alginate as its WT strain (Fig. 1), revealing that AlgL has a negative regulatory function. The observation that excess active AlgL reduced while excess inactive AlgL enhanced alginate yield (Fig. 1), indicated that AlgL negatively impacted alginate biosynthesis through its enzymatic activity.

The mechanism by which alginate polymer length is controlled is not completely understood. Nevertheless, recent studies have shown that alginate molecular mass was enhanced by higher copy numbers of the polymerization subunits Alg8 and Alg44. Furthermore, polymer length was shown to be influenced negatively by epimerization and positively by o-acetylation events^14^. However, the role of AlgL and its lyase activity in controlling polymer length (and composition) in *P. aeruginosa* has been poorly characterized. Previously, deletion of *algL* increased the molecular mass of alginates produced by *A. vinelandii and P. fluorescens*^33^,^46^; however, in the present study, deletion of *algL* in *P. aeruginosa* PDO300 did not affect alginate polymer length or its size distribution (Fig. 2), arguing that AlgL is not directly involved in controlling alginate molecular mass in this organism.

It is worth noting that other polysaccharide biosynthesis systems utilize extracellular polymer-degrading enzymes to control polymer length such as AlgA3 in alginate production by *A. vinelandii*^47^ and ExoK/ExsH in succinoglycan synthesis by *Sinorhizobium meliloti*^48^. However, this was not found to be the case for alginate production by *P. aeruginosa* PDO300 (Fig. S2).

The impact of AlgL on alginate composition in *P. aeruginosa* has not yet been investigated. While deletion of *algL* in *P. fluorescens* reduced epimerization degree^33^ manipulating AlgL copy number or enzymatic activity did not affect epimerization in *P. aeruginosa* (Table 1), indicating that epimerization occurs independently of AlgL. In contrast, deletion of *algL* significantly impaired O-acetylation (Table 1). Furthermore, since plasmid-borne expression of *algL* or *algL*_H202A_ in the *algL* mutant did not restore O-acetylation degree to WT levels (Table 1), we propose that native chromosomal expression of *algL* is critical for efficient O-acetylation. Consistent with this interpretation, located directly downstream of the *algL* gene is a weak promoter stationed directly upstream of *algI*, *J* and *F* genes which are essential for O-acetylation. Thus, it is plausible that disruption of the *algL* gene could adversely affect O-acetylation levels at a transcriptional and/or post-transcriptional level by interfering with mRNA stability and transcription and/or translation of these genes. Alternatively, chromosomal expression of *algL* could be important for proper localization and/or functioning of the *algI*, *J* and *F* gene products.

AlgL is thought to degrade misguided alginate in the periplasm. However, it was unclear whether or not it is a free periplasmic protein or a subunit of the multi-protein complex. In this study we provide several lines of evidence suggesting that AlgL is a free periplasmic protein dispensable for alginate production. Firstly, deletion of *algL* did not compromise alginate production (Fig. 1); affect polymer length control (Fig. 2), epimerization degree (Table 1) or stability of the biosynthesis complex (Fig. 3). Secondly, plasmid borne expression of *algL* (or *algL*_H202A_) in the *algL* mutant did not substantially affect alginate composition (Table 1). Thirdly, while previous reports^13^,^15^,^44^,^45^ demonstrated that deletion of structural elements of the biosynthesis complex (Alg44, K, X, E) abolished alginate production while destabilizing other subunits, in the present study, deletion of structural components did not affect AlgL stability (Fig. 3b), indicating that AlgL stability is independent of the integrity of the biosynthesis apparatus. Furthermore, we did not detect any interaction partner of AlgL in our protein interaction experiments (Fig. 4). These observations are consistent with findings of Farrell *et al*.^49^ which showed that AlgL displayed a surprising lack of stereospecificity to various alginates, further arguing that AlgL functioned exclusively to degrade misguided alginate in the periplasm without being directly involved in its polymerization, modification, and translocation/secretion process. Taken together, these results suggest that AlgL is a free periplasmic protein that is dispensable for alginate production.

However, since the *algL* gene is co-localized and presumably co-expressed with the other alginate biosynthesis genes, it is plausible that the AlgL protein could still be loosely associated with the biosynthesis complex. Although speculative, this possibility is supported by the observation that over-production of catalytically active AlgL (but not its catalytically inactive variant, AlgL_H202A_) interfered with polymer mass control (Fig. 2). Since there are hundreds of alginate biosynthesis complexes per cell as revealed by a recent immunogold-labelling study^50^, it is thus conceivable that the detrimental effect of excess copy numbers of AlgL on alginate production may have at least partially occurred due to an ‘over-crowding effect’ since additional copies of active AlgL could have altered the ratio of active AlgL with the number of active alginate production factories and/or factory subunits. Nevertheless, further work would be required to elucidate the mechanism by which excess active AlgL interfered with alginate polymer length control.

Polysaccharides and their respective degrading enzymes are thought to play important roles in biofilm attachment, growth and dispersal. For instance, a polysaccharide hydrolase, PslG facilitates biofilm dispersal by *Pseudomonas* species^51^. To examine if endogenously produced AlgL played a role in biofilm lifestyle, we performed microtiter plate experiments. We provide evidence suggesting that AlgL is not directly involved in biofilm attachment, growth or dispersal. Furthermore, the impact of manipulating AlgL copy number on alginate yield and molecular weight did not affect biofilm attachment, growth or dispersal. However, we observed a link between attachment efficiency and O-acetylation degree (Figs 5 and S5), supporting previous claims that O-acetylation enhances surface colonization, micro-colony formation while serving as a signal for cell-to-cell interaction^14^,^28^. Our evidence suggests that the dispersal stage of *P. aeruginosa* biofilms is also sensitive to alginate O-acetylation levels (Figs 5 and S5).

Although in this study AlgL was not found to be essential for alginate production, we propose that chromosomal expression of its gene, *algL*, could be important for the pathogenicity of *P. aeruginosa*. This is because chromosomal expression of *algL* boosted O-acetylation of alginate, a modification that has been implicated in protecting the pathogen from opsonic phagocytosis^52^ while enhancing activity of other virulence factors, such as the lipase, LipA which attacks host cell membranes^28^,^53^. Thus, chromosomal expression of *algL* could be important for the overall fitness of this pathogen especially during infection.

In conclusion, the outcomes of this work provide us with a better understanding of the biological function of a polysaccharide degrading enzyme in controlling alginate biosynthesis, molecular weight and composition. We provide compelling evidence that AlgL is likely to be a free periplasmic protein that is dispensable for alginate production. We also reveal that chromosomal expression of *algL* could be important for the pathogenicity of *P. aeruginosa* since native expression of *algL* was necessary for efficient O-acetylation of alginate, a modification which substantially enhances the fitness of this organism during infection. Overall, the knowledge acquired from this study is of significance for the development of strategies for alginate production and disease management.

## Methods

### Bacterial strains and growth conditions

Bacterial strains, plasmids and oligonucleotides of this investigation are listed in Table S1. Oligonucleotides were from Integrated DNA Technologies. *Escherichia coli* were grown in LB medium at 37 °C. pEX100T, pHERD20T and pFLP2 derived plasmids were introduced into *P. aeruginosa* with *E. coli* S17-1. *P. aeruginosa* strains were grown in LB or *Pseudomonas* Isolation (PI) medium and on PI agar (PIA) (Difco) at 37 °C. For *E. coli*, ampicillin (Amp) and gentamycin (Gm) were used at 100 and 10 μg/ml. For *P. aeruginosa*, carbenicillin (carb) and Gm were used at 300 μg/ml.

### Generation of isogenic marker free mutants

Isogenic marker free PDO300Δ*alg44*, *algX*, *algK*, *algE* and *alg8* mutants were made by homologous recombination using relevant pEX100TΔ*alg* gene-replacement plasmids^11^,^13^,^16^,^54^,^55^. A similar strategy was used to generate and confirm an isogenic marker-free PDO300Δ*algL* mutant (Supplementary Methods). Supplementary Methods summarize general manipulation of DNA.

### Generation of complementation plasmids

*algL* ORF flanked with *Nco*I and *Sma*I restriction sites was obtained by PCR using primer pairs algLNF(*Nco*I) and algLCR(*Sma*I). *algLH202A* and *algLx6his* genes flanked by the same sites were synthesized by Genscript. Above genes were ligated into corresponding sites of pHERD20T, generating pHERD20T:*algL*, pHERD20T:*algL*_*H202A*_ and pHERD20T:*algL*_*x6his*_.

### Alginate quantification

Alginate yields were determined^13^. Cells of an overnight culture (in LB) were washed twice with saline and suspended to an optical density (OD_600 nm_) of 30. 200 μl of suspended cells were spread onto a PIA carb 300 plate and incubated at 37 °C for 72 h. Biomass was suspended in saline and cells sedimented (7,500 g at 4 °C for 30 min). From supernatant, alginate was precipitated with 1 volume ice-cold isopropanol. Alginate and cells were lyophilized. Alginate was solubilized to 0.5% (wt/vol) in 0.05 M Tris-HCl–10 mM MgCl_2_ (pH 7.4). Samples were incubated with 15 μg of DNase I/ml and 15 μg of RNase A/ml at 37 °C for 6 h followed by 18 h treatment with Pronase E at 20 μg/ml. Solutions were dialyzed against 5 liters of ultrapure H_2_O for 48 h and alginate was precipitated with isopropanol and freeze-dried for quantification and uronic acid analysis, using algal alginate as a standard^13^,^56^.

In this study alginate quantification for each strain (treatment) was performed in three biological replicates.

### Analysis of alginate molecular mass

Alginate molecular weight and polydispersity were characterized by size exclusion chromatography-multi-angle laser light scattering (SEC-MALLS) analysis^14^. DAWN-EOS multi-angle laser light scattering detector with a laser at 690 nm (Wyatt Technology Corp., Santa Barbara, CA, USA); Waters 2410 refractive index monitor). Purified samples were dissolved in 0.1 M NaNO_3_ (2 mg/mL) and allowed to hydrate fully by incubating at room temperature overnight. Immediately prior to analysis, samples were pre-heated at 80 °C for 5 min, injected (100 μL) and eluted with 0.1 M NaNO_3_ (0.7 mL/min, 60 °C) from two columns (TSK-Gel G5000PWXL and G4000PWXL, 300 × 7.8 mm, Tosoh Corp.) connected in series. ASTRA software (version 6.1.2.84, Wyatt Technology Corp.) and dn/dc of 0.150 mL/g was used for determining weight-average molecular weights (*M*_*r*_) and number-average molecular weights (Mn) and polydispersity index (PI) via the fraction Mw/Mn. In the case of a perfectly monodisperse (homogeneous) polymer PI value equals 1.0.

### Determination of alginate composition

Degree of O-acetylation and epimerization of alginate was established by ^1^H-nuclear magnetic resonance (NMR) spectroscopy^14^. The spectra were recorded at 90 °C with a JEOL 270 NMR spectrometer (6.34 T) operating at 270 MHz for proton. Samples were prepared as described^57^. The chemical shifts were expressed in ppm downfield from the signal for 3-(trimethylsilyl) propanesulfonate. We determined the composition of the different de-acetylated alginate samples and their acetylation degree by integration of the ^1^H-NMR signals. For alkaline de-acetylation 30 ml of 1% alginates in saline solution were treated with 12 ml of 1 M NaOH in 65 °C for 30 min and neutralized with 12 ml of 1 M HCl. Treated samples were then dialyzed against 5 L of distilled water for 48 h and then freeze-dried.

### Pulling down of hexahistidine tagged AlgL

Strains PDO300Δ*algL*(pHERD20T:*algL*_*x6his*_) and PDO300Δ*algL*(pHERD20T:*algL*) were grown for 72 h on PIA carb 300+/− 0.05% (w/v) arabinose. Biomass was re-suspended in saline and cells were washed thrice with PBS, suspended in Buffer A (supplemented with 0.1 mg/ml lysozyme and 0.1 mg/ml DNAse A), and lysed by sonication. [Composition of Buffer A: 100 mM phosphate buffer, 150 mM NaCl, 5 mM imidazole, 5 mM EDTA, 1x Roche EDTA-free protease inhibitor, 10% (v/v) glycerol].

Hexahistidine-tagged AlgL and its potential co-interacting proteins were purified with cOmplete His-Tag Purification Resin (Roche). Envelope fractions were solubilized in Buffer B [Buffer A supplemented with 0.2% (v/v) Triton X-100T, for 1 h on ice] and incubated with pre-equilibrated Roche Resin for 1 h on ice. Unbound proteins were removed by centrifugation (10,000 g for 10 s) and impurities were removed by four washes with Buffer B. To elute histagged-AlgL, resin was incubated with Buffer C (Buffer B containing 400 mM imidazole) on ice for 10 min then centrifuged.

### Immunoblot analysis

Envelope fractions were prepared (Supplementary Methods) and protein concentrations were assessed by Bradford Assay (Bio-Rad Protein Assay Kit, Bio-Rad Laboratories, Inc.). Proteins were run on SDS-PAGE and transferred to a nitrocellulose membrane using iBlot® Dry Blotting system (Invitrogen). Membranes were washed thrice with TBST (Tween 20 0.1% v/v) and blocked with TBST + 5% w/v BSA for 1 h at 25 °C. Membranes were washed and probed at 4 °C overnight with rabbit polyclonal antibody (Genscript) in TBST + 1% w/v BSA. Anti-Alg44, -AlgE, -AlgK, and -AlgL antibodies were used at 1:10,000 and Anti-AlgX was used at 1:7,000 dilutions. Membranes were probed with goat-anti-rabbit antibody conjugated to horse radish peroxidase (HRP) for 1 h at 25 °C (1:10,000 in TBST + 1% w/v BSA). Membranes were incubated for 5 min with substrate and image developed. Anti-histag-HRP antibodies were used to detect AlgLx6his (Abcam).

### Solid surface attachment and dispersal assays

Biofilm attachment assays were performed^58^. Strains were grown for 24 h in PI medium containing Carbenicillin (300 μg/ml) and 0.5% (w/v) of arabinose. Cultures were standardized to an O.D_600 nm_ of 2.0 using respective culture supernatants. To 8 wells of sterile microtiter plate, 100 μL of standardized cultures was added, and plate was incubated for 2 h at 37 °C. Unbound-cells were removed by washing with water and bound-cells quantified by crystal violet staining (Supplementary Methods). Attachment efficiency was inferred from absorbance.

For dispersal assays, 24 h pre-cultures (in PI) were standardized to O.D_600 nm_ of 0.20 with PI medium containing Carbenicillin at 300 μg/ml. 100 μL was added to each of 4 wells of a 96-well microtiter plate and incubated for 72 h at 37 °C. Unbound-cells were removed by washing, and strains were incubated with 200 μL of saline supplemented with 0.5% w/v arabinose for 1 h. After a x10,000 fold dilution, cells were spread on PIA carb plates and colony forming units counted. Remaining biomass (non-dispersed cells) was measured by crystal violet staining (Supplementary Methods). In the latter case, absorbance represented the biofilm biomass at 72 h of growth.

For microtiter plate assays, average (±standard error) of cellular attachment efficiency, biofilm biomass at 72 h, and biofilm dispersal efficiency of each strain was determined from eight, four and four biological replicates, respectively.

### Statistical analysis

Data that were normally distributed (Shapiro-Wilk test: P > 0.05) were analyzed using ANOVA with multiple LSD test for multiple comparison between treatments. However, data that were not normally distributed (Shapiro-Wilk test: P < 0.05) were analyzed using non-parametrical ANOVA following by Bonferroni (Dunn) t tests for multiple comparison between treatments. All analyses were done using SAS 9.13. Rejection level was set at α < 0.05. Reported values were means ± standard error (SE).

### Alginate lyase plate assay

Detection of alginate lyase activity in cultures grown on solid media was performed, with modifications^59^. Strains were grown on PIA medium for 72 h. Biomass was suspended in saline and cells were pelleted by centrifugation. Supernatants were filter-sterilized and 100 μl of cell-free re-suspensions were dropped onto alginate plates for 24 h at 37 °C. Plate composition: LB supplemented with 1% w/v agarose and 0.1% w/v sodium alginate (Sigma Aldrich, USA). Plates were flooded overnight with 10% w/v cetylpyridinium chloride. Clearings indicated alginate degradation. Commercial alginate lyase (Sigma Aldrich, USA) was used as positive control.

## Additional Information

**How to cite this article**: Wang, Y. *et al*. Biological function of a polysaccharide degrading enzyme in the periplasm. *Sci. Rep*. **6**, 31249; doi: 10.1038/srep31249 (2016).

## Supplementary Material

Supplementary Information

## Figures and Tables

**Table 1:** Alginate composition as measured by H^3^-NMR.

Strain	F_G_	F_M_	F_GM/MG_	F_MM_	F_GG_	AC%
WT	0.28	0.72	0.28	0.44	0	70
ΔalgL	0.35	0.65	0.35	0.3	0	17
ΔalgL + algL	0.36	0.64	0.36	0.28	0	20
ΔalgL + algLH202A	0.36	0.64	0.36	0.28	0	19

Alginate samples, obtained from strains grown on PIA medium containing Carbenicillin (300 μg/ml) and arabinose inducer at 0.5% (w/v), were analysed by NMR to investigate their level of epimerization and O-acetylation. *F*_*G*_: molar fraction of guluronate residues in alginate chain. *F*_*M*_: mole fraction of mannuronate residues in alginate chain. *F*_*GM/MG*_, *F*_*MM*_and *F*_*GG*_: molar fraction of GM/MG, MM and GG diads. AC%: degree of O-acetylation. Strains are identified as follows: WT = PDO300(pHERD20T), ΔalgL = PDO300Δ*algL*(pHERD20T), ΔalgL + algL = PDO300Δ*algL*(pHERD20T:*algL*), and ΔalgL + algLH202A = PDO300Δ*algL*(pHERD20T:*algL*_H202A_).
