# Supplementary material for: Biological function of a polysaccharide degrading enzyme in the periplasm
Source: Sci Rep. 2016 Nov 8;6:31249. doi: 10.1038/srep31249 (PMC5099689; doi:10.1038/srep31249)
Supplement: Supplementary Information [file srep31249-s1.pdf]

Biological function of a polysaccharide degrading enzyme in the periplasm

Yajie Wang[1], M. Fata Moradali[1], Ali Goudarztalejerdi[3], Ian M. Sims[4], Bernd H. A. Rehm[1,2*]

Affiliations

[1] Institute of Fundamental Sciences, Massey University, Palmerston North, New Zealand

[2] MacDiarmid Institute for Advanced Materials and Nanotechnology, Massey University, Palmerston North, New Zealand

[3] Department of Pathobiology, School of Paraveterinary Science, Bu-Ali Sina University, Hamadan, Iran

[4] The Ferrier Research Institute, Victoria University of Wellington, Lower Hutt, Wellington, New Zealand

[*] B.Rehm@massey.ac.nz (Institute of Fundamental Sciences and MacDiarmid Institute for Advanced Materials and Nanotechnology, Massey University, Private Bag 11222, Palmerston North 4442, New Zealand)

Supplementary Methods

Generation and confirmation of *algL* mutant

To generate an isogenic marker-free PDO300Δ*algL* mutant, the gene-replacement plasmid pEX100TΔ*algLΩGm*^{R} was constructed and introduced into *P. aeruginosa* PDO300 (Figure S1). To construct this plasmid, two regions of the *algL* gene were synthesized. These regions algLN and algLC were 352 and 449 bp in length, and corresponded to nucleotides 12-363 and 644-1093 bp of *algL* ORF. At the 3' end of algLN and at the 5' end of algLC, *Bam*HI restriction sites were introduced. These DNA fragments were digested with *Bam*HI and then ligated with a FRT-*aacC1*-FRT cassette (a 1,100 bp *Bam*HI fragment from pPS856) into *Sma*I (a blunt-cutter) linearized pEX100T plasmid yielding pEX100TΔ*algLΩGm*^{R}. (FRT = flip-recombinase recognition sites; *aacC1* = a gentamycin resistance marker). pEX100TΔ*algLΩGm*^{R} was introduced into *P. aeruginosa* PDO300 by transconjugation. Integration of gentamycin resistance marker and subsequent deletion of 280 bp of *algL* open reading frame (ORF) was verified by PCR using primers algLupXout and algLdownXout.

Manipulation of DNA

Deoxynucleoside triphosphates, Taq, and Platinum Pfx polymerases were from Invitrogen. Restriction enzymes were from New England Biolabs. DNA was cloned using CloneJET PCR Cloning kit (Thermo Scientific, USA). DNA was verified by sequencing using an ABI310 automatic sequencer.

Isolation of envelope fraction

Envelope fractions were isolated [1]. Cells were lysed by lysozyme treatment (for 20 min on ice in 100 mM phosphate buffer, pH 7.5, 150 mM NaCl, 0.1 mg/mL of lysozyme and 0.1 mg/ml of DNAase I) followed by sonication. Cell debris was removed (15,000 g for 45 min at 4 °C) and envelope fractions were sedimented (100,000 g for 90 min at 4 °C).

Crystal violet staining

To each well of microtitre plate, 150 µL of crystal violet solution (0.1% w/v in water) was added and then incubated for 10 min. Unbound stain was removed by washing with water. Plates were dried and bound crystal violet was solubilized in 200 µL of DMSO and absorbance at 595 nm was measured.

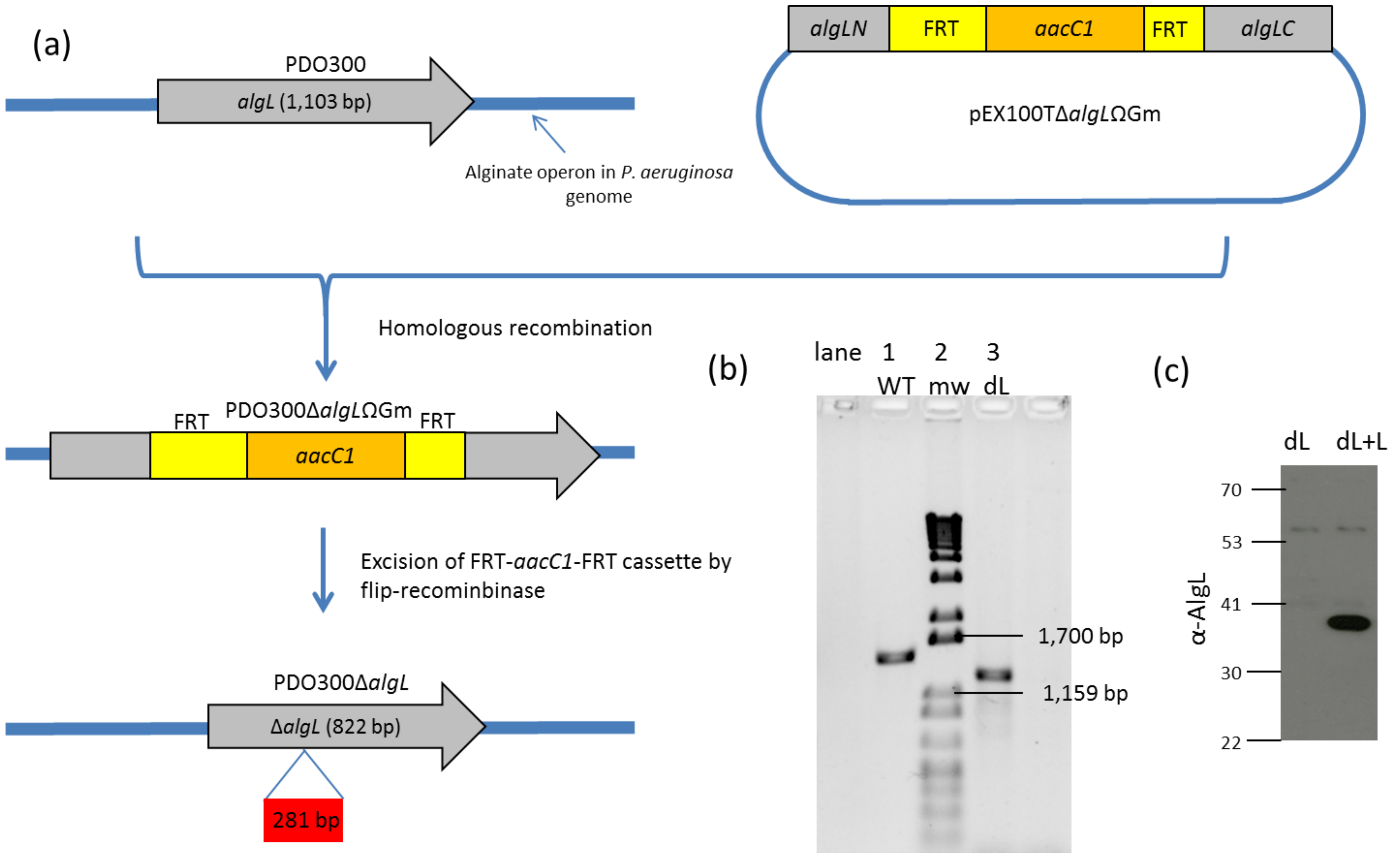
(a)
PDO300
algL (1,103 bp)
Alginate operon in P. aeruginosa genome
algLN
FRT
aacC1
FRT
algLC
pEX100TΔalgLΩGm
Homologous recombination
FRT
PDO300ΔalgLΩGm
FRT
aacC1
Excision of FRT-aacC1-FRT cassette by flip-recominbinase
PDO300ΔalgL
ΔalgL (822 bp)
281 bp
(b)
lane 1 2 3
WT mw dL
1,700 bp
1,159 bp
(c)
dL dL+L
70
53
41
30
22
α-AlgL

Figure S1. Generation and confirmation of PDO300Δ*algL* and its complemented strain (**a**) A schematic detailing the generation and confirmation of PDO300Δ*algL* mutant. To generate PDO300Δ*algL*, a gene replacement plasmid pEX100TΔ*algL*Gm was constructed and introduced into PDO300 for homologous recombination, replacing 281 bp of *algL* gene with an FRT-*aacC1*-FRT cassette. This cassette contains a gentamycin resistance marker (*aacC1*) flanked by flp-recombinase recognition sites (FRT). A flp-recombinase encoding plasmid, pFLP2,was introduced to remove the FRT-*aacC1*-FRT cassette. Excision of the cassette and deletion of 281 bp of *algL* gene (Red Rectangle) in the PDO300Δ*algL* mutant was confirmed by colony PCR. (**b**) PDO300 and PDO300Δ*algL* were confirmed by colony PCR using primers algLupXout and algLdownXout which flank the *algL* gene in the chromosome. PCR products were subject to agarose gel electrophoresis and stained by ethidium bromide. PCR products from PDO300 (Lane 1, WT) and PDO300Δ*algL* (Lane 3, dL) are shown at expected molecular weights of 1,465 and 1,349 bp, respectively. Lane 2 = DNA MW Ladder (mw). (**c**) Immunoblot of envelope fractions to verify the absence and presence of AlgL protein in the *algL* mutant [PDO300Δ*algL*(pHERD20T)] and its complemented strain [PDO300Δ*algL*(pHERD20T:*algL*)] when grown on PIA containing Carbenicillin (300 µg/ml) and arabinose (0.5% w/v).

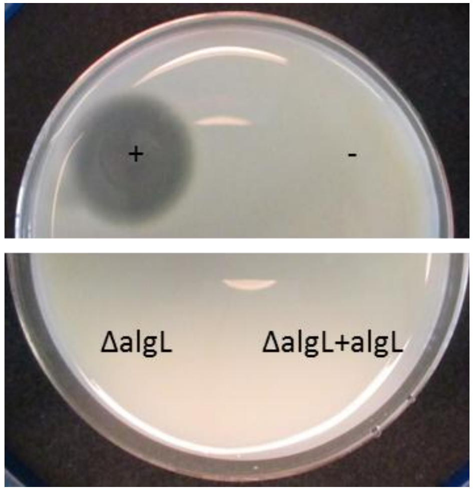

Figure S2. Alginate lyase assay of strains grown on solid media. Strains were grown in presence of 0.5% (w/v) arabinose on PIA (72 h) medium containing Carbenicillin (300 µg/ml). Biomass of biofilms was re-suspended in saline and then filter-sterilized. 100 µL of cell-free re-suspensions were dropped onto alginase assay plates and incubated for 24 h at 37 °C. Plates were flooded with 10 % w/v cetylpyridinium chloride for overnight incubation. Clearings are indicative of alginate degradation. Commercial alginate lyase (Sigma Aldrich, USA) and autoclaved saline were used as positive (+) and negative (-) controls. Strains identified as follows: ΔalgL = PDO300Δ*algL*(pHERD20T), and ΔalgL+algL = PDO300Δ*algL*(pHERD20T:*algL*).

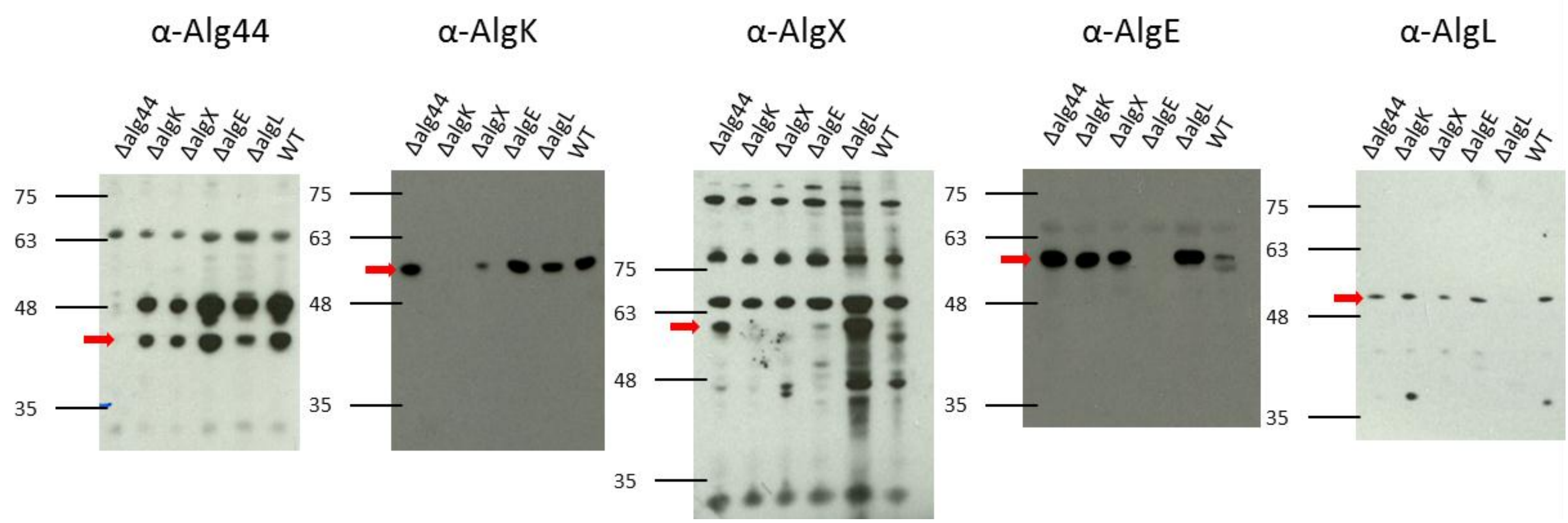

Figure S3. Full length immunoblots for detection of components of the alginate biosynthesis apparatus in various *alg* mutants. Shown are immunoblots of envelope fractions of various strains using anti-Alg antibodies (*from left*: α-Alg44, α-AlgK, α-AlgX, α-AlgE and α-AlgL) to detect specific components of biosynthesis complex. Red arrows highlight target proteins at expected molecular weight. Strains are identified as follows: Δalg44 = PDO300Δ*alg44*, ΔalgK = PDO300Δ*algK*, ΔalgX = PDO300Δ*algX*, ΔalgE = PDO300Δ*algE*, ΔalgL = PDO300Δ*algL*, and WT = *P. aeruginosa* PDO300. MW markers are shown in kDa.

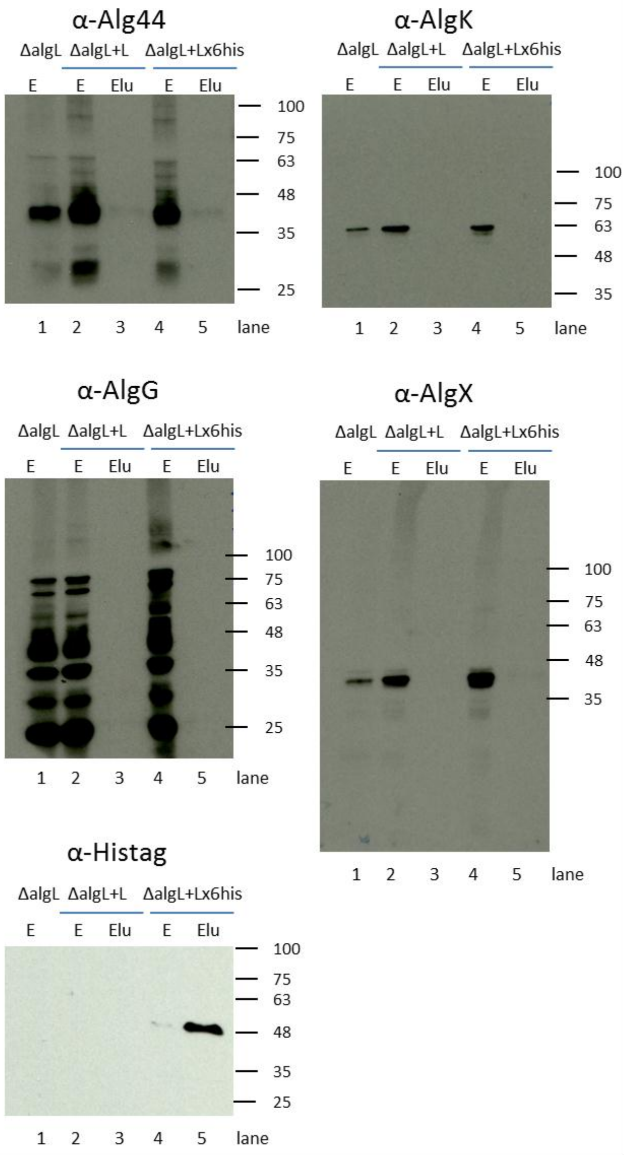
α-Alg44
ΔalgL ΔalgL+L ΔalgL+Lx6his
E E Elu E Elu
100 75 63 48 35 25
1 2 3 4 5 lane
α-AlgK
ΔalgL ΔalgL+L ΔalgL+Lx6his
E E Elu E Elu
100 75 63 48 35
1 2 3 4 5 lane
α-AlgG
ΔalgL ΔalgL+L ΔalgL+Lx6his
E E Elu E Elu
100 75 63 48 35 25
1 2 3 4 5 lane
α-AlgX
ΔalgL ΔalgL+L ΔalgL+Lx6his
E E Elu E Elu
100 75 63 48 35
1 2 3 4 5 lane
α-Histag
ΔalgL ΔalgL+L ΔalgL+Lx6his
E E Elu E Elu
100 75 63 48 35 25
1 2 3 4 5 lane

Figure S4. Full length immunoblots showing the detection of hexahistidine-tagged AlgL and its interaction partners. Strains were grown on PIA medium supplemented with 300 µg/ml of Carbenicillin (except for PDO300Δ*algL*, lane 1) and 0.05% (w/v) arabinose for 72 h at 37 °C. Envelope (E) fractions were prepared and histagged-AlgL and its co-interacting proteins were purified with c0mplete His-Tag Purification Resin (Roche). Envelope (E) and eluted (Elu.) fractions were run on SDS-PAGE, transferred to nitrocellulose membrane and subject to immunoblot with various antibodies (*identified above each panel*: α-Alg44, α-AlgK, α-AlgG, α-AlgX and α-Histag), as described in methods section. In each panel, Lane 1 = Envelope fraction of PDO300Δ*algL*. Lanes 2 and 3 = Envelope (E) and eluted (Elu.) fractions of PDO300Δ*algL*(pHERD20T:*algL*), respectively. Lanes 4 and 5 = Envelope (E) and eluted (Elu.) fractions of PDO300Δ*algL*(pHERD20T $algL_{x6his}$), respectively.

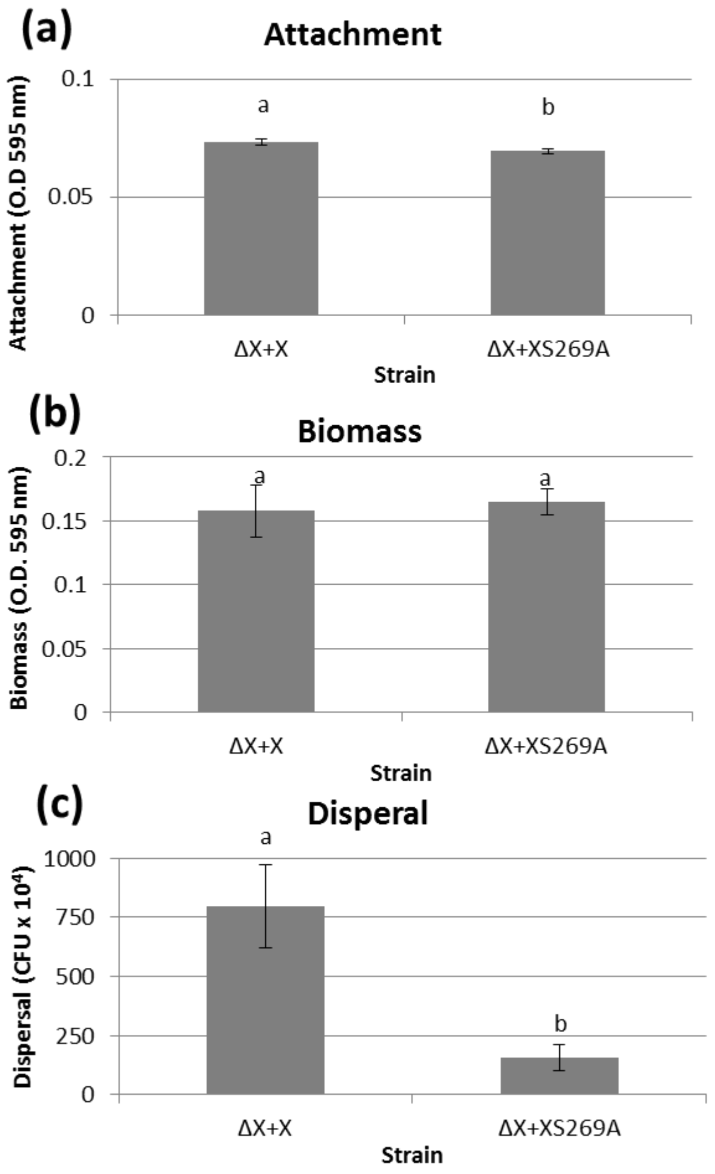
(a)
Attachment
Attachment (O.D 595 nm)
0.1
0.05
0
a
b
ΔX+X
ΔX+XS269A
Strain
(b)
Biomass
Biomass (O.D. 595 nm)
0.2
0.15
0.1
0.05
0
a
a
ΔX+X
ΔX+XS269A
Strain
(c)
Disperal
Dispersal (CFU x 10⁴)
1000
750
500
250
0
a
b
ΔX+X
ΔX+XS269A
Strain

Figure S5. Effect of alginate O-acetylation on cell attachment, biofilm biomass and dispersal efficiency. Attachment efficiencies, biofilm biomasses and dispersal efficiencies were analyzed in a microtiter plate assay. (**a**) Mean attachment efficiencies ± SE (n =6) at 2 h, as determined by crystal violet staining. Attachment efficiencies are expressed as absorbances at 595 nm. (**b**) Mean biomass of biofilms ± SE (n =4) at 72 h, as determined by crystal violet staining. (**c**) Mean dispersal efficiencies ± SE (n=6) at 72 h expressed as colony forming units (CFU x 10^4). In panels (**a**), (**b**) and (**c**) different letters displayed above columns indicate statistically significant differences (two-way, t-test, $p < 0.05$) in attachment efficiency, biofilm biomass and dispersal efficiency, respectively. In panels (**a**), (**b**) and (**c**), strains ΔalgX+algX and ΔalgL+algXS269A are identified as PDO300Δ*algX*(pBBR1MCS-5:*algX*) and PDO300Δ*algX*(pBBR1MCS-5:*algXS269A*), respectively. These strains produce O-acetylated and non-acetylated forms of alginate, respectively.

Table S1. Strains, plasmids and oligonucleotides used in this study.

Strains, plasmids or oligonucleotides	Description	Source
Strains		
P. aeruginosa		
PDO300	*mucA22 isogenic mutant derived from PAO1*	[2]
PDO300Δ*algL*	Isogenic *algL* deletion mutant derived from PDO300	This study
PDO300Δ*alg44*	Isogenic *alg44* deletion mutant derived from PDO300	[3]
PDO300Δ*algK*	Isogenic *algK* deletion mutant derived from PDO300	[4]
PDO300Δ*algX*	Isogenic *algX* deletion mutant derived from PDO300	[5]
PDO300Δ*algE*	Isogenic *algE* deletion mutant derived from PDO300	[6]
E. coli		
TOP10	*E. coli* cloning strain	Invitrogen
S17-1	thi-1 proA hsdR17 (rK- mK- recA1; tra gene of plasmid RP4 integrated in chromosome	
Plasmids		
pEX100TΔ*algL*ΩGm	Ap^r Cb^r Gm^r; vector pEX100T with *Sma*I-inserted *algL* deletion construct	This study
pHERD20T	Ap^r Cb^r, vector with arabinose inducible promoter (*araC*-P_{BAD} cassette)	[7]
pHERD20T:*algL*	*algL* ORF (*Nco*I-*Sma*I) inserted into pHERD20T	This study
pHERD20T:$algL_{H202A}$	*algL* ORF with H202A mutation (*Nco*I-*Sma*I) inserted into pHERD20T	This study
pHERD20T:$algL_{x6his}$	*algL* ORF with C-terminal hexahistdine tag (*Nco*I-*Sma*I) inserted into pHERD20T	This study
pFLP2	Ap^r Cb^r; broad-host-range vector encoding Flp recombinase	This study
Oligonucleotides		
*algL*N(NcoISDNd)	CGATCCATGGGAGGAGATAATCGCTATGAAACGTCCCACCTGATCCGTATCG	This study
*algL*C(SmaI)	CGCTAT CCCGGGCACTCAACTTCCCCCTTCGCGGCTG	This study
algL upXout	GATCGAGCAGTCGAAAGCCGTCGATACC	This study
algL downXout	CGTTTTCCTCTGGTGTTGCCTAAG	This study

Supplementary References

1. Wang, Y., Hay, I. D., Rehman, Z. U. & Rehm, B. H. A. Membrane-anchored MucR mediates nitrate-dependent regulation of alginate production in *Pseudomonas aeruginosa*. *Appl. Microbiol.Biotechnol.* **99**, 7253-7265 (2015).
2. Mathee, K. *et al.* Mucoid conversion of *Pseudomonas aeruginosa* by hydrogen peroxide: a mechanism for virulence activation in the cystic fibrosis lung. *Microbiology-Sgm* **145**, 1349-1357 (1999).
3. Remminghorst, U. & Rehm, B. H. A. Alg44, a unique protein required for alginate biosynthesis in *Pseudomonas aeruginosa*. *Febs Lett.* **580**, 3883-3888 (2006).
4. Rehman, Z. U., Wang, Y. J., Moradali, M. F., Hay, I. D. & Rehm, B. H. A. Insights into the assembly of the alginate biosynthesis machinery in *Pseudomonas aeruginosa*. *Appl. Environ. Microbiol.* **79**, 3264-3272 (2013).
5. Gutsche, J., Remminghorst, U. & Rehm, B. H. A. Biochemical analysis of alginate biosynthesis protein AlgX from *Pseudomonas aeruginosa*: purification of an AlgX-MucD (AlgY) protein complex. *Biochimie* **88**, 245-251 (2006).
6. Hay, I. D., Rehman, Z. U. & Rehm, B. H. A. Membrane topology of outer membrane protein AlgE, which is required for alginate production in *Pseudomonas aeruginosa*. *Appl. Environ. Microbiol.* **76**, 1806-1812 (2010).
7. Qiu, D., Damron, F. H., Mima, T., Schweizer, H. P. & Yu, H. D. P-BAD-based shuttle vectors for functional analysis of toxic and highly regulated genes in *Pseudomonas* and *Burkholderia* spp. and other bacteria. *Appl. Environ. Microbiol.* **74**, 7422-7426 (2008).
